# An online nomogram based on bimodal ultrasound images for preoperative diagnosis of cytologically indeterminate thyroid nodules

**DOI:** 10.3389/fendo.2026.1838372

**Published:** 2026-06-29

**Authors:** Wenwu Lu, Di Zhang, Xin Wu, Wenbo Ding, Xiang Xie, Lei Hu, Lei Ye, Wang Zhou, Chaoxue Zhang

**Affiliations:** 1 Department of Ultrasound, The First Affiliated Hospital of Anhui Medical University (Jixi Campus), Hefei, Anhui, China; 2 Department of Ultrasound, The First Affiliated Hospital of Anhui Medical University (Gaoxin Campus), Hefei, Anhui, China; 3 Department of Ultrasound, Affiliated Hospital of Integration Chinese and Western Medicine with Nanjing University of Traditional Chinese Medicine, Nanjing, China; 4 Department of Ultrasound, The Second Affiliated Hospital of Anhui Medical University, Hefei, Anhui, China; 5 Department of Ultrasound, The First Affiliated Hospital of University of Science and Technology of China (USTC), Division of Life Sciences and Medicine, University of Science and Technology of People’s Republic of China, Hefei, Anhui, China

**Keywords:** molecular testing, radiomics, thyroid nodules, transfer learning, ultrasound

## Abstract

**Background:**

Accurately distinguishing benign from malignant indeterminate thyroid nodules is essential to avoid unnecessary diagnostic surgeries. This study aims to develop an online nomogram model that enables precise preoperative assessment of malignancy risk in indeterminate thyroid nodules (ITNs) following fine-needle aspiration, thereby helping to reduce unnecessary thyroidectomies.

**Methods:**

Patients with thyroid nodules were recruited from five centers and divided into retrospective training, independent testing, and prospective validation cohorts. Radiomics features and deep learning features were extracted from both B-mode ultrasound (BMUS) and strain elastography ultrasound (SEUS) images for each patient. Malignancy-associated features were selected to construct BMUS and SEUS signature scores, respectively. Multivariate regression analysis was performed on all variables to develop and visualize a comprehensive model for diagnosing benign and malignant ITNs. The model was further evaluated for discrimination, calibration, and clinical usefulness.

**Results:**

Multimodal imaging features, genetic testing, and elastography levels were identified as key biological markers for diagnosing ITNs. The nomogram model built with these variables demonstrated strong performance. The area under the receiver operating characteristic curve was 0.907 (95% CI: 0.877-0.931) in the training set, 0.885 (95% CI: 0.821-0.932) in the external test set, and 0.860 (95% CI: 0.762-0.929) in the prospective validation set. Compared to clinical and individual scoring models, the nomogram demonstrated superior performance and calibration.

**Conclusions:**

The proposed nomogram accurately diagnoses ITNs and holds promise for reducing unnecessary diagnostic thyroidectomies in clinical practice.

## 1 Introduction

Thyroid nodules (TNs) are a common clinical issue, with a prevalence of up to 65% in the general population, including both benign and malignant thyroid nodules (1, 2). Although most nodules are benign, approximately 7% to 15% carry a malignant potential, typically thyroid cancer (3). Recent data suggest a decline in thyroid cancer incidence, while incidence-based mortality has shown a slight but statistically non-significant increase (4). Early detection and accurate diagnosis of malignant thyroid nodules are essential for reducing unnecessary surgeries (5). Despite the generally favorable prognosis for thyroid cancer, some patients with papillary thyroid cancer present with neck lymph node metastasis at the time of diagnosis, necessitating surgical intervention (6). Early detection and treatment can reduce cancer spread and improve patient quality of life (7). Therefore, accurately diagnosing the benign or malignant nature of thyroid nodules is critical for clinical management.

Thyroid ultrasound is the optimal initial method for evaluating thyroid nodules and plays a crucial role in their management and diagnosis (8). Common imaging modalities include B-mode ultrasound and elastography (9). B-mode ultrasound provides structural and morphological information, while elastography assesses tissue stiffness, which is crucial for distinguishing benign from malignant nodules (10). A previous multiparametric ultrasound study suggested that multimodal ultrasound may improve risk prediction for thyroid nodules with indeterminate or uncertain malignant potential (11). However, to further determine the malignancy of highly suspicious thyroid nodules, FNA is often required, as recommended by guidelines from the American College of Radiology (ACR) and the European Thyroid Association (12, 13). FNA is a widely used, minimally invasive method for diagnosing thyroid nodules, with a high accuracy rate (14, 15).

However, FNA has limitations. Unlike histopathological diagnosis following excision, FNA is confined to cytomorphological features (16). According to the Bethesda System for Reporting Thyroid Cytopathology, cytologically indeterminate thyroid nodules (ITNs) generally include Bethesda categories III–V, for which the risk of malignancy varies substantially and clinical management remains challenging (17). When cytology is indeterminate, molecular testing can further stratify malignancy risk and support decisions regarding surveillance or surgery (18, 19). However, repeated FNA or diagnostic lobectomy is still frequently required to obtain a definitive diagnosis, which may increase patient burden and lead to unnecessary surgical intervention in benign cases (17, 20). Therefore, a non-invasive and clinically applicable approach for malignancy risk assessment in ITNs is urgently needed.

In recent years, artificial intelligence (AI) technologies, including machine learning, deep learning, and transfer learning, have rapidly advanced in the field of ultrasound imaging diagnostics (21). AI-based methods can automatically extract high-dimensional imaging features and provide quantitative assessments that may reduce observer dependence in thyroid ultrasound interpretation (22, 23). Machine learning and deep learning also hold significant potential for application in thyroid cytology and histopathology (24). The International Head and Neck Scientific Group suggests that integrating molecular research and imaging techniques into clinical practice can comprehensively assess the malignancy risk of Bethesda III nodules (25). Nevertheless, existing AI-based models for thyroid nodule diagnosis still have several limitations. Many models rely mainly on single-modality ultrasound images or conventional B-mode features, and few specifically focus on cytologically indeterminate nodules (26). In addition, most available models lack integration with molecular testing, external validation, prospective evaluation, or user-friendly tools for bedside risk estimation, which limits their generalizability and clinical translation (27, 28).

To address these limitations, this study aimed to develop and validate an online nomogram for the preoperative diagnosis of ITNs by integrating bimodal ultrasound information from B-mode ultrasound and strain elastography with molecular testing data. Unlike previous models that primarily relied on single-modality imaging or imaging features alone, our model combines radiomics features, deep learning features, elastography assessment, and genetic mutation status. We further evaluated the model in independent testing and prospective validation cohorts and developed an online tool to facilitate individualized malignancy risk estimation. This approach may help reduce repeat FNA and unnecessary diagnostic thyroidectomy while supporting more precise clinical decision-making for patients with ITNs.

## 2 Materials and methods

### 2.1 Patients and grouping

This was a multicenter diagnostic study with retrospective model development and external testing, followed by prospective validation. Consecutive patients with cytologically indeterminate thyroid nodules who underwent thyroid ultrasound, fine-needle aspiration, molecular testing, and subsequent surgical pathological confirmation were screened from five centers. The sampling method was consecutive sampling during the predefined study periods. No formal sample-size calculation was performed; the final sample size was determined by the number of eligible patients with complete clinical, ultrasound, molecular, and pathological data.

This study was approved by the Institutional Ethics Committee. The retrospective study was granted a waiver for informed consent, while all participants in the prospective study provided written informed consent. The retrospective cohort included 470 patients from Centers 1–4 treated between January 2022 and April 2025, serving as the training set, along with 143 patients from Centers 5 treated between January 2022 and January 2023 as the external test set. The prospective validation cohort consisted of 76 patients recruited from Centers 1 and 2 between February and April 2025. Center definitions were as follows: Center 1, The First Affiliated Hospital of Anhui Medical University (Jixi Campus); Center 2, The First Affiliated Hospital of Anhui Medical University (Gaoxin Campus); Center 3, The Second Affiliated Hospital of Anhui Medical University; Center 4, The First Affiliated Hospital of USTC; and Center 5, Affiliated Hospital of Integration Chinese and Western Medicine with Nanjing University of Traditional Chinese Medicine. All patients were categorized into benign or malignant groups based on surgical pathology results. Detailed inclusion and exclusion criteria for study participants are provided in the **Supplementary S1** and **Supplementary Figure 1**. A total of 679 patients were ultimately included in this study.

### 2.2 Clinical pathological data and ultrasound image collection

Baseline clinical pathological information, including gender, age, tumor size, lesion location, Bethesda category, molecular testing results, elastography level, and surgical pathology results, was collected from hospital information systems. All thyroid ultrasound images were retrieved from the Picture Archiving and Communication System (PACS) of all participating centers. BMUS and SEUS examinations were performed by radiologists with more than five years of experience. Standardized scanning and FNA protocols were implemented across all participating centers, and image preprocessing was performed to minimize inter-device variability. Elastography levels were assessed using a four-point scoring system, with colors ranging from red to blue indicating soft to hard tissue, respectively (29). FNA was performed under ultrasound guidance by physicians with over ten years of experience in interventional ultrasound. After appropriate processing, the aspirated tissue was sent to the pathology department for cytological diagnosis and molecular testing. Molecular testing for BRAF, RAS, and TERT mutations was performed on the residual FNA specimens prior to surgery. Detailed descriptions of the elastography ultrasound and FNA procedures are provided in the **Supplementary S2**.

### 2.3 Image segmentation and feature extraction

**Figure 1:**
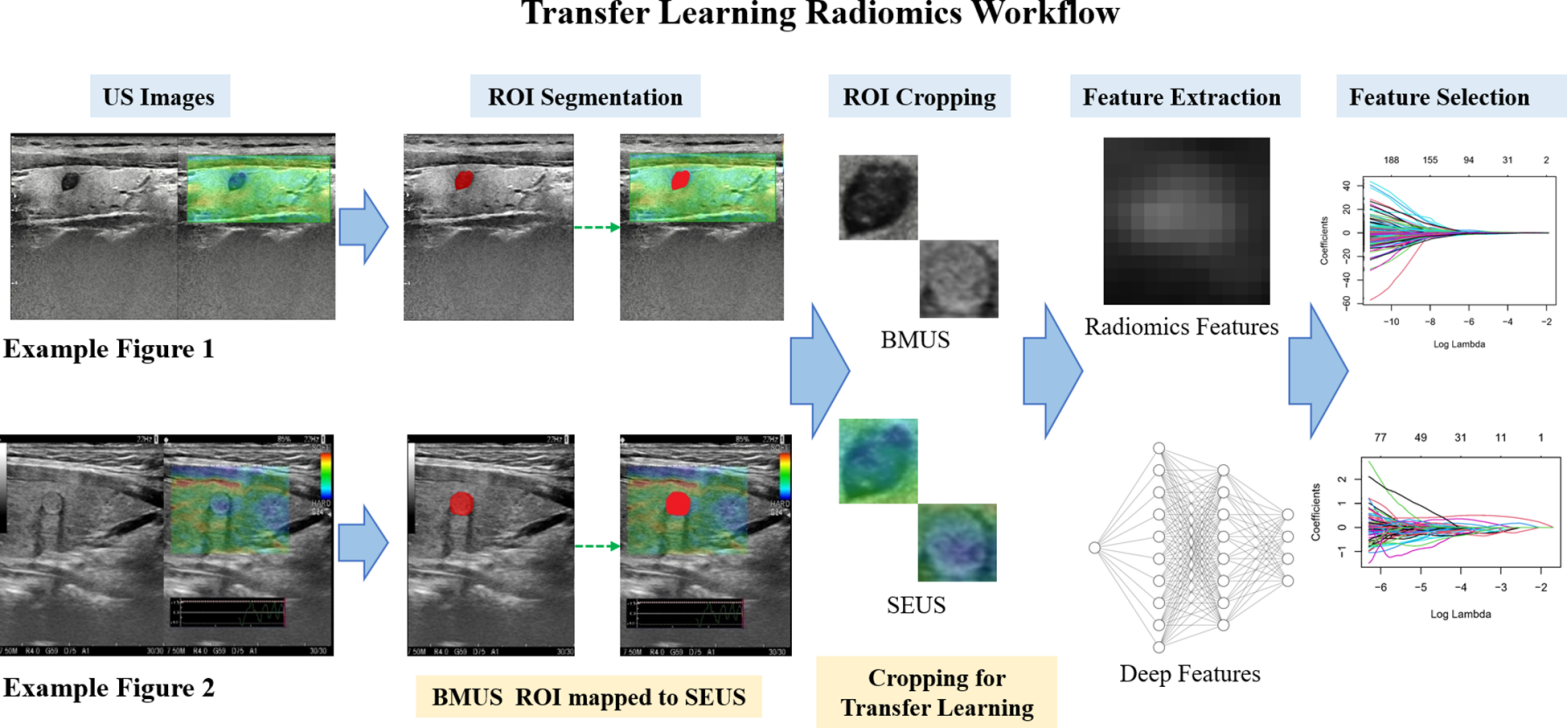
Workflow of the transfer learning radiomics process in this study. US, ultrasound; ROI, region of interest; BMUS, B-model ultrasound; SEUS, Strain Elastography ultrasound.

All images were reviewed by a radiologist with at least five years of experience in thyroid imaging. For each nodule, the image with the largest diameter and best quality was selected from the PACS for further analysis (detailed in **Supplementary S2**). The radiologist was blinded to all patient information beyond the ultrasound images. Each ultrasound image consisted of a BMUS image on the left and an elastography image on the right. Using the ITK-SNAP software (version 3.8.0, available at http://www.itksnap.org) , the radiologist manually outlined Region of Interest 1 (ROI 1) on the left-side BMUS image, then mapped the ROI 1 mask onto the right-side elastography image to obtain Region of Interest 2 (ROI 2). Gray-scale BMUS and elastography images were cropped from the respective ROIs for transfer learning. Radiomics features from both ROIs were extracted using the Pyradiomics library in Python (30). The DenseNet201 model was utilized as the pre-trained model for extracting deep learning features (31). **Figure 1** illustrates the workflow of this study. The detailed feature extraction process is described in the **Supplementary S3**. To ensure reproducibility, images from 50 randomly selected patients were re-segmented by another radiologist with five years of experience. Features with an interclass correlation coefficient (ICC) of less than 0.8 were considered poorly reproducible and excluded from the analysis. The ICC scatter plot results are shown in **Supplementary Figure 2**.

### 2.4 Feature selection and score construction

Radiomics features and deep learning features from the same imaging modality were combined to construct BMUS and SEUS scores after undergoing the following feature selection steps. Before score construction, all extracted features were standardized using z-score normalization based on the mean and standard deviation of the training cohort. The same normalization parameters were then applied to the external test and prospective validation cohorts. Feature reproducibility was first assessed using the ICC, and only features with ICC values greater than 0.8 were retained. Redundant features were subsequently removed to reduce feature collinearity. Finally, the least absolute shrinkage and selection operator (LASSO) logistic regression with five-fold cross-validation was used to select the most predictive features and determine their corresponding coefficients (32).

The BMUS score was calculated as a linear combination of the selected BMUS-derived radiomics and deep learning features weighted by their LASSO coefficients. This score was designed to quantify structural, morphological, and textural information from gray-scale B-mode ultrasound images. Similarly, the SEUS score was calculated using selected strain elastography-derived radiomics and deep learning features, reflecting stiffness-related heterogeneity within thyroid nodules. The general calculation formula was as follows:

Score = β0 + β1 × Feature 1 + β2 × Feature 2 + … + βn × Feature n.

Where β0 represents the intercept, and β1 to βn represent the coefficients of the selected features. Detailed steps for feature selection and score construction are provided in the **Supplementary S4**. The BMUS and SEUS scores were then entered as continuous variables into the multivariate logistic regression model to construct the final nomogram.

### 2.5 Model construction

Univariate analysis was performed in the training set to identify risk factors associated with nodule malignancy, with a significance threshold of p < 0.05. Subsequently, all independent risk factors were entered into a stepwise backward multivariate regression analysis to determine the final effective variables, leading to the construction of a nomogram model. To visualize the model, a nomogram was generated, and a Nomo-score was calculated for each patient. Additionally, prediction models based on clinical ultrasound factors and the two scores were developed to compare the performance differences between models.

### 2.6 Statistical analysis

Statistical analyses were performed using R software (version 4.4.1) and SPSS software (version 26.0). ROC curve plotting and analysis were carried out using MedCalc software (version 20.1). The optimal cutoff value for the Nomo-score was identified by maximizing the Youden index. The area under the ROC curve (AUC) was used to assess the discriminative ability of the models, and the DeLong test was applied to compare differences in AUCs between models. Calibration of the nomogram was evaluated using calibration curves and the Hosmer-Lemeshow test. Decision curve analysis (DCA) and clinical impact curve (CIC) were employed to assess the clinical utility of the nomogram at different thresholds. All statistical significance levels were two-sided, with a p < 0.05 and a 95% confidence interval (CI).

## 3 Results

### 3.1 Clinicopathological characteristics

**Table 1** presents the patient information and clinicopathological baseline characteristics in both the training and external validation sets. Malignant ITNs accounted for 57.9% (272/470), 56.6% (81/143), and 64.5% (49/76) of cases in the three datasets respectively. As shown in **Table 2**, univariate analysis of clinical and ultrasound factors in the training set identified four variables with statistically significant differences between benign and malignant groups: elastography grade, genetic mutation status, BMUS score, and SEUS score. These four variables were subsequently incorporated into multivariate logistic regression analysis to establish the nomogram. Additionally, we developed a clinical model using only two variables: elastography score and genetic mutation status (**Table 3**). The clinical model demonstrated AUC values of 0.810 (95% CI: 0.772-0.845), 0.754 (95% CI: 0.675-0.822), and 0.746 (95% CI: 0.633-0.839) in the training set, test set, and validation set respectively.

**Table 1:** Clinicopathological characteristics in the training and test datasets.

Characteristic	Training set (n = 470)	Test set (n = 143)	Validation set (n = 76)
Pathology			
benign	198 (42.1)	62 (43.4)	27 (35.5)
malignant	272 (57.9)	81 (56.6)	49 (64.5)
Sex			
male	117 (24.9)	34 (23.8)	10 (13.2)
female	353 (75.1)	109 (76.2)	66 (86.8)
Age, mean ± SD, years	45.7 ± 11.2	46.5 ± 10.4	44.4 ± 13.0
Tumor location			
left	197 (41.9)	67 (46.9)	23 (30.3)
right	261 (55.5)	73 (51.0)	3 (3.9)
isthmus	12 (2.6)	3 (2.1)	50 (65.8)
Tumor size, mean ± SD, mm	11.8 ± 7.0	11.8 ± 8.9	10.2 ± 6.0
Bethesda category			
III	107 (22.8)	37 (25.9)	22 (28.9)
IV	77 (16.4)	24 (16.8)	13 (17.1)
V	286 (60.8)	82 (57.3)	41 (54.0)
Elastography level			
1	27 (5.7)	7 (4.9)	2 (2.6)
2	223 (47.5)	76 (53.1)	31 (40.8)
3	172 (36.6)	50 (35.0)	27 (35.5)
4	48 (10.2)	10 (7.0)	16 (21.1)
Genetic mutation			
negative	269 (57.2)	92 (64.3)	36 (47.4)
positive	201 (42.8)	51 (35.7)	40 (52.6)
Pathology type			
nodular goiter	41 (8.7)	18 (12.6)	9 (11.8)
thyroid adenoma	92 (19.6)	27 (18.9)	15 (19.7)
other benign types	65 (13.8)	17 (11.9)	3 (4.0)
PTC	258 (54.9)	74 (51.7)	48 (63.2)
FTC	10 (2.0)	5 (3.5)	1 (1.3)
MTC	2 (0.4)	0 (0)	0 (0)
NIFTP	3 (0.6)	2 (1.4)	0 (0)

Data represent the count of patients and percentages when not specified. Other benign types include hyaline degeneration, calcification, cysts and follicular epithelial hyperplasia. PTC, papillary thyroid carcinoma; FTC, follicular thyroid carcinoma; MTC, medullary thyroid carcinoma; NIFTP, non-invasive follicular thyroid neoplasm with papillary-like nuclear features.

**Table 2:** Univariate analysis in the training set.

Characteristic	Training set (n = 470)		
	Malignant (n=272)	Benign (n=198)	*p* value
Sex			0.477
male	71 (26.1)	46 (23.2)	
female	201 (73.9)	152 (76.8)	
Age, mean ± SD, years	45.4 ± 11.4	46.1 ± 11.0	0.485
Tumor location			0.563
left	109 (40.1)	88 (44.5)	
right	155 (57.0)	106 (53.5)	
isthmus	8 (2.9)	4 (2.0)	
Tumor size, mean ± SD, mm	11.4 ± 5.8	12.4 ± 8.4	0.172
Bethesda category			0.759
III	65 (23.9)	42 (21.2)	
IV	45 (16.5)	32 (16.2)	
V	162 (59.6)	124 (62.6)	
Elastography level			<0.001*
1	3 (1.1)	24 (12.1)	
2	93 (34.2)	130 (65.7)	
3	133 (48.9)	39 (19.7)	
4	43 (15.8)	5 (2.5)	
Genetic mutation			<0.001*
negative	104 (38.2)	165 (83.3)	
positive	168 (61.8)	33 (16.7)	
BMUS score, median (IQR)	0.77(0.58-0.88)	0.38(0.18-0.55)	<0.001*
SEUS score, median (IQR)	0.79(0.58-0.93)	0.34(0.17-0.56)	<0.001*

Data represent the count of patients and percentages when not specified. BMUS, B-mode ultrasound; SEUS: strain elastography ultrasound; SD, standard deviation; IQR, interquartile range. *p < 0.05.

**Table 3:** The clinical model and the combined model were constructed using multivariate regression analysis in the training set.

Intercept and variable	Clinical model			Comprehensive model		
	β	Odds ratio (95% CI)	p value	β	Odds ratio (95% CI)	p value
Intercept	−3.294			−2.370		
Elastography level	1.218	3.381 (2.381-4.802)	<0.001*	0.681	1.976 (1.309-2.981)	0.001*
Genetic mutation	1.732	2.322 (3.516-9.078)	<0.001*	1.411	4.102 (2.340-7.190)	<0.001*
BMUS score	NA	NA	NA	1.016	2.763 (1.767-4.320)	<0.001*
SEUS score	NA	NA	NA	0.603	1.827 (1.469-2.273)	<0.001*

CI, confidence interval; BMUS, B-mode ultrasound; SEUS, strain elastography ultrasound. *p < 0.05.

### 3.2 Feature selection and score construction

From each modality image, 464 radiomics features and 128 deep learning features were extracted. After evaluating the features using ICC, features with ICC > 0.8 were retained for further analysis. Statistical analysis was performed on the training set, and the LASSO algorithm was used to identify the features most relevant to predicting ITNs. The LASSO algorithm ultimately selected 14 features from BMUS images and 11 features from SEUS images to construct the BMUS and SEUS scores, respectively. The selected BMUS features consisted of radiomics features and deep learning features, including first-order statistics, shape-based features, wavelet-transformed texture features, and six DenseNet201-derived deep learning features. These features mainly reflected the gray-scale intensity distribution, lesion shape, margin-related morphology, and intranodular textural heterogeneity on B-mode ultrasound images.

The selected SEUS features included first-order statistics, gray-level dependence matrix features, wavelet-based texture features, neighborhood gray-tone difference matrix features, and three DenseNet201-derived deep learning features. These features were considered to reflect stiffness distribution and elastography-based heterogeneity within thyroid nodules. The BMUS and SEUS scores were calculated by multiplying each selected feature by its corresponding LASSO coefficient and summing these weighted features with the intercept. The complete calculation formulas and weighting coefficients are provided in **Supplementary S5**, and the feature selection process is illustrated in **Supplementary Figure 3**.

The BMUS score demonstrated AUCs of 0.826 (95% CI: 0.788-0.859) in the training set, 0.774 (95% CI: 0.696-0.840) in the test set, and 0.732 (95% CI: 0.618-0.828) in the external validation set for predicting malignancy. Similarly, the SEUS score achieved AUCs of 0.849 (95% CI: 0.813-0.880), 0.831 (95% CI: 0.759-0.888), and 0.757 (95% CI: 0.645-0.848) in the training, test, and external validation sets respectively.

### 3.3 Construction and evaluation of the nomogram

**Figure 2:**
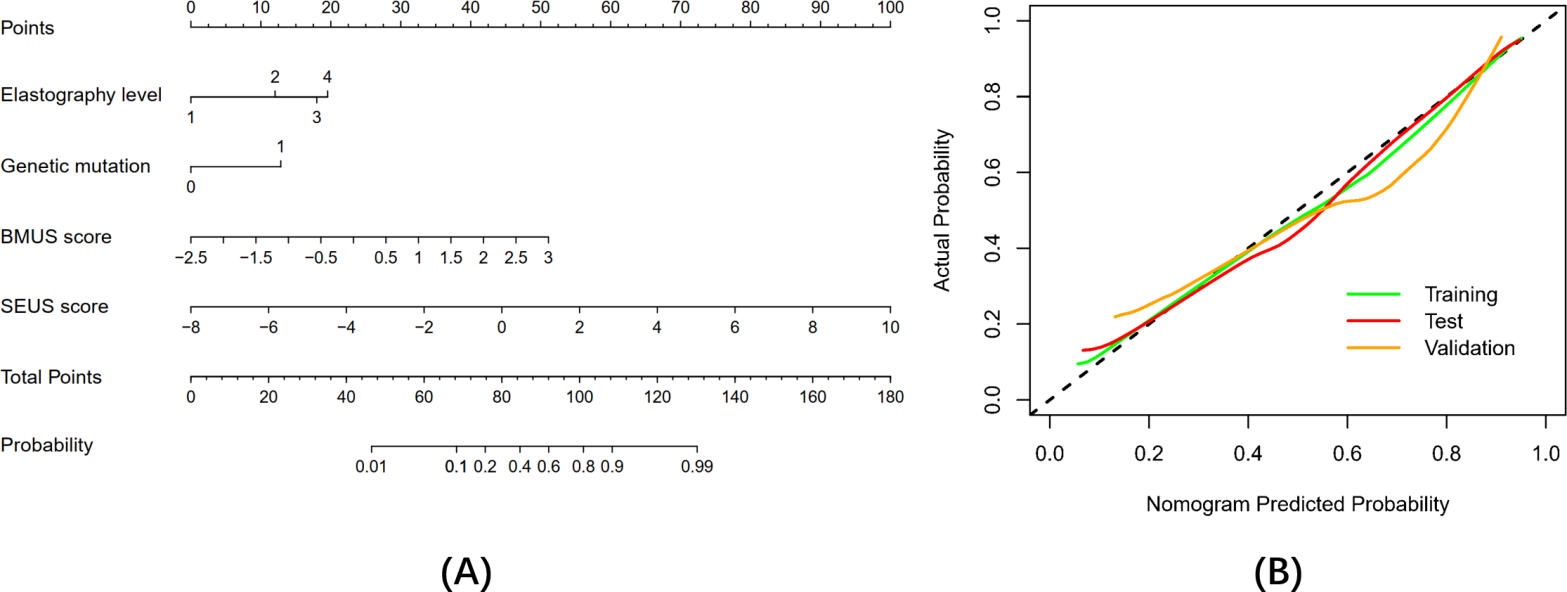
Construction and calibration evaluation of the nomogram. **(A)** The comprehensive model is visualized as a nomogram to predict the malignancy probability of indeterminate thyroid nodules. **(B)** Calibration performance of the nomogram in the training and external validation sets. The p-values in the training and validation sets are 0.224, 0.538 and 0.898, respectively. BMUS, B-mode ultrasound; SEUS, strain elastography ultrasound.

**Figure 3:**
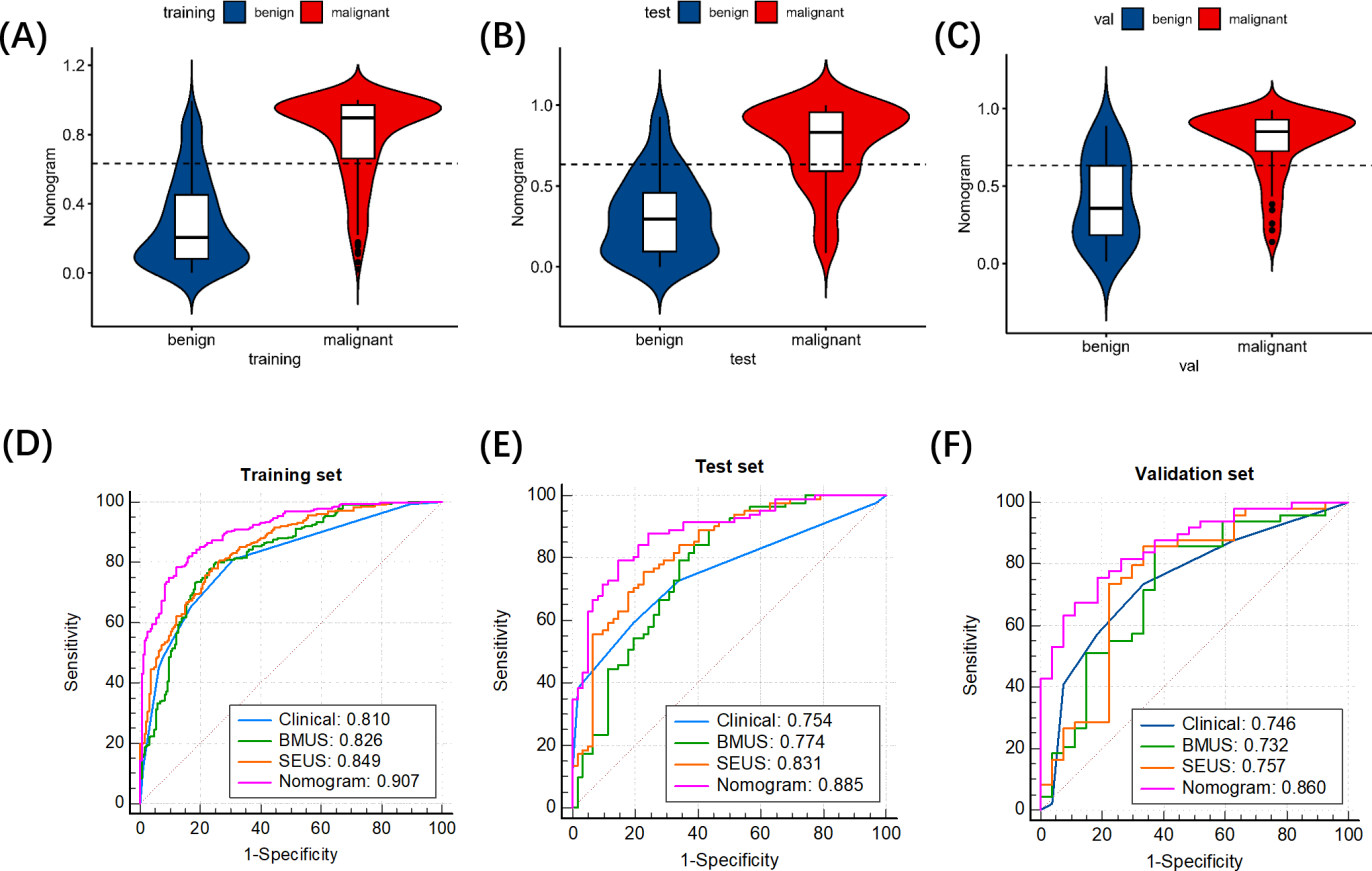
Performance of the comprehensive nomogram model. **(A–C)** represent the data distribution of the nomogram in the training and test sets, while demonstrating the risk classification performance at the optimal cutoff value of 0.633. **(D–F)** show the receiver operating characteristic curves for predicting indeterminate thyroid nodule status in the training set, test set, and validation set, respectively.

Multivariable logistic regression demonstrated that elastography grade, genetic mutations, BMUS score, and SEUS score were statistically significant predictors for ITNs. We constructed a nomogram incorporating these predictive factors (**Table 3**). **Figure 2A** presents the visualized nomogram of the model. The calibration curves in **Figure 2B** indicate excellent agreement between predicted and observed outcomes for ITN classification in both training and test sets. Hosmer-Lemeshow tests yielded p-values of 0.224, 0.538, and 0.875 for the calibration curves in the training set, test set, and validation set, respectively. The optimal cutoff value of 0.633 for the composite model score was determined using the maximum Youden index. **Figures 3A, C**) demonstrate effective discrimination between benign and malignant cases at this cutoff across all datasets. Model performance metrics are illustrated in **Figures 3D, F** and **Table 4**. The composite model achieved AUC values of 0.907 (95% CI: 0.877-0.931) in the training set, 0.885 (95% CI: 0.821-0.932) in the test set, and 0.860 (95% CI: 0.762-0.929) in the external validation set. We developed an online dynamic nomogram (https://webnomogram.shinyapps.io/ITNapp/) to facilitate clinicians’ calculation of malignancy probability for cytologically indeterminate thyroid nodules post-FNA.

**Table 4:** The clinical model and the combined model were constructed using multivariate regression analysis in the training set.

Model performance	Clinical model				BMUS score				SEUS score			Comprehensive model		
	Training set	Test set	Validation set	Training set		Test set	Validation set	Training set		Test set	Validation set	Training set	Test set	Validation set
AUC (95% CI)	0.810 (0.772 -0.845)	0.754 (0.675 -0.822)	0.746 (0.633 - 0.839)	0.826 (0.788 -0.859)		0.774 (0.696 -0.840)	0.732 (0.618-0.828)	0.849 (0.813 -0.880)		0.831 (0.759 -0.888)	0.757 (0.645-0.848)	0.907 (0.877 -0.931)	0.885 (0.821-0.932)	0.860 (0.762-0.929)
Sen	0.813	0.728	0.735	0.677		0.617	0.940	0.691		0.691	0.877	0.849	0.852	0.766
Spe	0.687	0.661	0.667	0.838		0.742	0.334	0.823		0.823	0.514	0.793	0.758	0.744
PPV	0.781	0.737	0.800	0.852		0.758	0.718	0.843		0.836	0.766	0.849	0.821	0.846
NPV	0.727	0.651	0.583	0.654		0.597	0.755	0.660		0.671	0.698	0.793	0.797	0.647
PLR	2.595	2.151	2.422	4.186		2.392	1.441	3.910		3.897	1.885	4.101	3.521	3.473
NLR	0.273	0.411	0.406	0.386		0.516	0.196	0.375		0.375	0.248	0.190	0.195	0.306
ACC	0.760	0.699	0.711	0.745		0.671	0.724	0.747		0.748	0.748	0.826	0.811	0.765

BMUS, B-mode ultrasound; SEUS: strain elastography ultrasound; AUC, area under the receiver operating characteristic curve; CI, confidence interval; Sen, Sensitivity; Spe, Specificity; PPV, positive predictive value; NPV, negative predictive value; PLR, positive likelihood ratio; NLR, negative likelihood ratio; Acc, accuracy.

**Figure 4:**
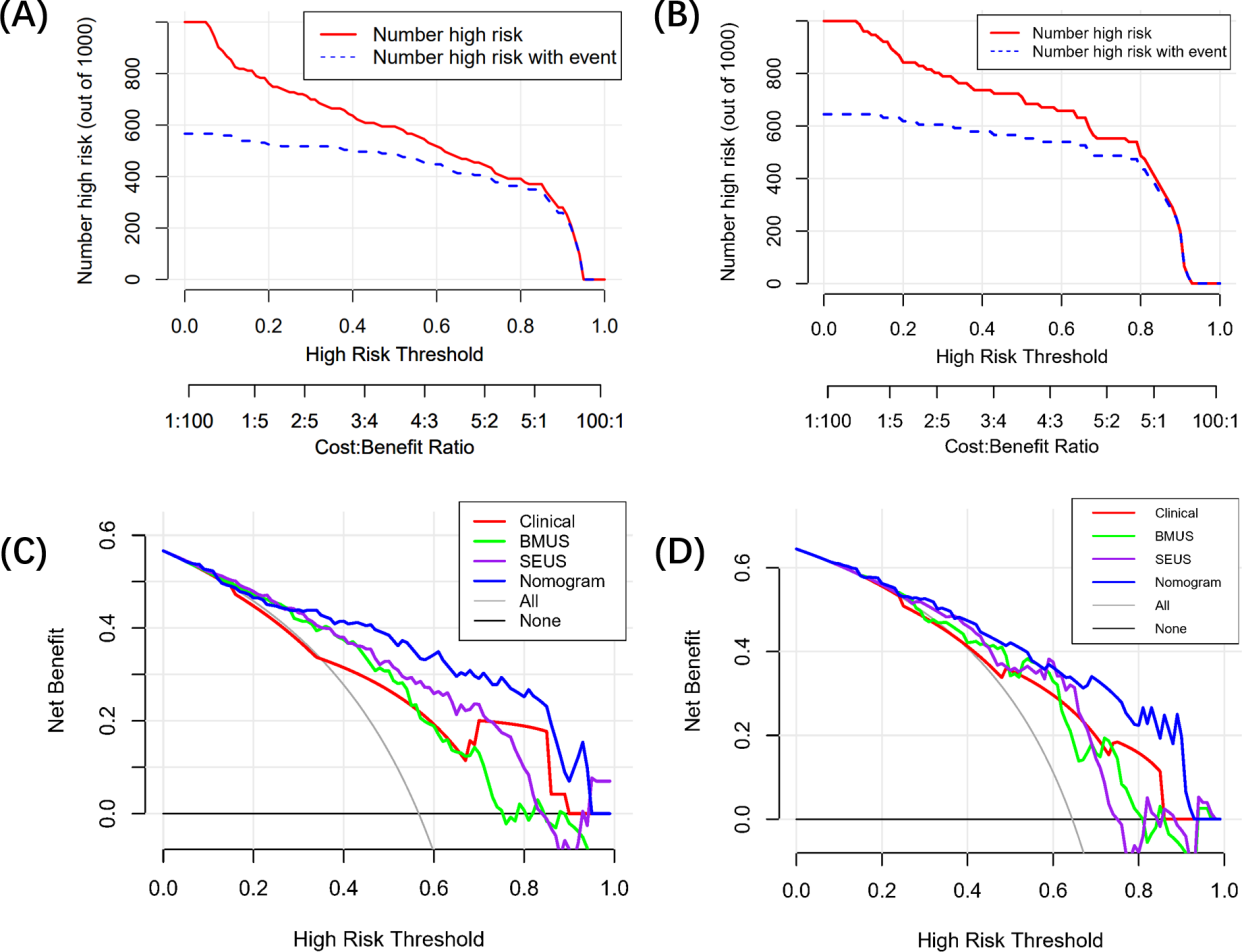
**(A)** Clinical impact curve of the integrated model in the test set and **(B)** validation set, with the x-axis representing probability thresholds and cost-benefit ratios, and the y-axis indicating the number of high-risk individuals per thousand. **(C)** Decision curve analysis of different models in the test set and **(D)** validation set, where the x-axis corresponds to risk thresholds and the y-axis represents net benefits.

Furthermore, clinical impact curves enabled assessment of high-risk population proportions at different probability thresholds, with cost-benefit analysis supporting clinical decision-making. In both test and validation sets (**Figures 4A, B**), when the threshold probability exceeded 0.7, the number of high-risk cases closely approximated the actual number of thyroid cancer cases, indicating strong clinical validity. Decision curve analysis (DCA) curves (**Figures 4C, D** demonstrated greater net benefit when using the composite model for ITN diagnosis compared to alternative approaches. DeLong’s test results in **Table 5** confirmed statistically superior performance of the composite model over clinical models, BMUS score, and SEUS score individually.

**Table 5:** Comparison of AUC values between different models.

Model comparison	Training set		Test set		Validation set	
	AUC	*p* value	AUC	*p* value	AUC	*p* value
Clinical model vs. Comprehensive	0.810 vs. 0.907	<0.001*	0.754 vs. 0.885	<0.001*	0.746 vs. 0.860	0.035*
Clinical model vs. BMUS	0.810 vs. 0.826	0.537	0.754 vs. 0.774	0.696	0.746 vs. 0.732	0.871
Clinical model vs. SEUS	0.810 vs. 0.849	0.102	0.754 vs. 0.831	0.117	0.746 vs. 0.757	0.906
BMUS vs. SEUS	0.826 vs. 0.849	0.236	0.774 vs. 0.831	0.201	0.732 vs. 0.757	0.753
BMUS vs. Comprehensive	0.826 vs. 0.907	<0.001*	0.774 vs. 0.885	0.002*	0.732 vs. 0.860	0.023*
SEUS vs. Comprehensive	0.849 vs. 0.907	<0.001*	0.831 vs. 0.885	0.031*	0.757 vs. 0.860	0.042*

AUC, area under the receiver operating characteristic curve; BMUS, B-mode ultrasound; SEUS, strain elastography ultrasound; *p < 0.05.

### 3.4 Subgroup analysis and case examples

**Figure 5:**
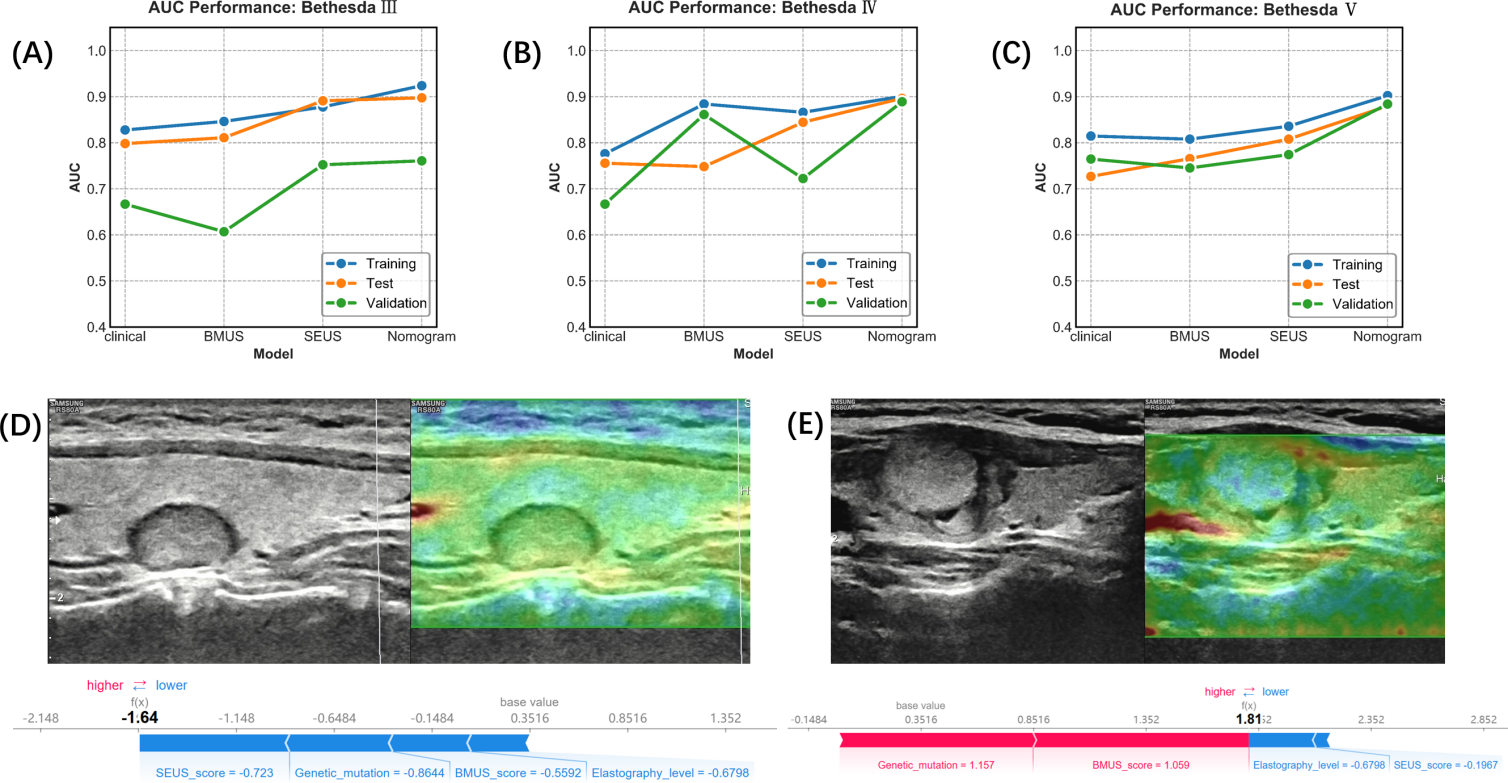
**(A-C)** demonstrate the predictive performance of each model across three subgroups and different cohorts for distinguishing benign from malignant thyroid nodules. **(D)** A 21-year-old female patient whose ultrasound images revealed extracapsular extension inferiorly, with histopathological examination suggesting an oncocytic adenoma. The SHAP force plot demonstrates all four features provided negative contributions, resulting in a final predicted value f(x) of -1.64, which was lower than the model’s baseline value of 0.35, leading to a benign prediction. **(E)** A 36-year-old female patient whose ultrasound images showed superior extracapsular extension, with genetic testing revealing RAS gene mutation and histopathology confirming invasive follicular carcinoma. The SHAP force plot indicates the BMUS score and genetic mutation features provided strong positive contributions, yielding a final predicted value f(x) of 1.81 that exceeded the baseline value, resulting in a malignant prediction.

The data from each dataset were stratified into three subgroups (Bethesda categories III, IV, and V) to evaluate the predictive performance of each model across different subgroups. The training set included 107 Bethesda III, 77 Bethesda IV, and 286 Bethesda V nodules; the test set included 37, 24, and 82; the validation set included 22, 13, and 41, respectively. As illustrated in **Figures 5A–C**, the nomogram model demonstrated superior diagnostic efficacy in all three subgroups. To explore the decision-making process of the integrated model for individual cases, we employed Shapley Additive Explanations (SHAP) force plots to interpret the contribution of each feature to the model’s predictions. **Figures 5D, E**) present two thyroid nodule cases diagnosed as Bethesda category IV (oncocytic follicular neoplasms) following FNA. Despite both cases exhibiting an elasticity grade of 2, their histopathological outcomes were markedly different. The SHAP force plots elucidate the model’s decision-making process: the base value represents the model’s average predicted probability without feature-specific influences, while red arrows indicate positive contributions and blue arrows denote negative contributions. The length of each arrow reflects the magnitude of the feature’s impact on the final prediction score, f(x). The model’s accurate classification of these nodules may help reduce diagnostic lobectomies for indeterminate cases and provide clinicians with valuable guidance for surgical decision-making.

## 4 Discussion

In this study, we collected thyroid nodule cases from five centers and constructed an online nomogram for the preoperative diagnosis of cytologically indeterminate thyroid nodules. By integrating BMUS score, SEUS score, elastography level, and genetic mutation status, the proposed model provided individualized malignancy risk estimation after indeterminate FNA cytology. Clinically, this tool may help identify patients who are more likely to benefit from surgery while supporting more conservative management or follow-up in patients predicted to have a low malignancy risk.

Ultrasound is currently the preferred imaging method for evaluating thyroid nodules (18). Risk stratification systems based on ultrasound features are widely used in the management of thyroid nodules and guide the decision to FNA (33). However, traditional two-dimensional ultrasound has limited value in distinguishing between benign and malignant thyroid nodules (34). SEUS evaluates tissue elasticity by assessing tissue displacement caused by compression (35). According to Saleem et al., SEUS is a non-invasive diagnostic method with high diagnostic accuracy, significantly enhancing the preoperative diagnosis of malignant thyroid nodules (36). In the evaluation of Bethesda IV nodules, Latia et al. incorporated elastography into the American College of Radiology Thyroid Imaging Reporting and Data System (ACR TI-RADS), achieving a sensitivity of 90.62% in detecting thyroid cancer (37). However, some studies have pointed out that SEUS grading is subjective, and less experienced physicians require more expertise (38, 39). Therefore, there is a need to objectify and quantify elastography to better describe the state of thyroid nodules.

AI technology excels at automatically recognizing complex patterns and providing quantitative assessments of ultrasound imaging data, offering significant potential to help physicians achieve more accurate and reproducible results (21). Liu et al. used radiomics to study multimodal images, including BMUS and SEUS, and found that multimodal images provided more information, improving the accuracy of lymph node metastasis assessment in patients with papillary thyroid carcinoma (40). Wang et al. applied multimodal ultrasound radiomics to diagnose ACR TI-RADS category 4–5 thyroid nodules, demonstrating that a combined diagnostic model incorporating clinical features, conventional ultrasound, and elastography had higher diagnostic performance (41). Hu et al. developed a deep learning model based on elastography and conventional ultrasound images of the perinodular area, improving the radiological diagnosis accuracy of thyroid cancer (42). Compared with previous ultrasound radiomics or deep learning studies, our study has several distinctive features. First, many previous models focused on general thyroid nodules, ACR TI-RADS 4–5 nodules, papillary thyroid carcinoma, or lymph node metastasis rather than cytologically indeterminate thyroid nodules (43, 44). Second, most previous studies relied mainly on conventional ultrasound, elastography, or imaging features alone, whereas our model integrated radiomics features, deep learning features, elastography assessment, and molecular testing data (26, 28, 45). Third, beyond retrospective model development, we further evaluated the model in an independent external test cohort and a prospective validation cohort and developed an online nomogram to facilitate individualized bedside risk estimation. These characteristics may improve the clinical applicability and translational potential of the proposed model.

While FNA is highly sensitive and accurate for diagnosing thyroid nodules, managing ITNs remains a clinical challenge (20, 46). For thyroid nodules classified as Bethesda III, IV, or V based on FNA, some studies recommend gene testing, as gene mutations can serve as predictive markers for thyroid malignancy in indeterminate nodules, significantly improving the detection rate of malignancies (47, 48). Gong et al. found that combining ultrasound-guided FNA with BRAF gene testing increased the malignancy detection rate during thyroid surgery and reduced unnecessary diagnostic surgeries for benign and non-significant lesions (49). The recently published third edition of the Bethesda system also emphasizes the importance of molecular testing, recommending for the first time that molecular testing be performed on Bethesda V nodules to aid in surgical decision-making (17). In our study, gene mutations were significantly associated with malignant thyroid nodules and could serve as important variables in diagnosing ITNs, based on univariate and multivariate analyses. However, nodule size and location were not found to be significant in this study. Sakajiri et al. suggested that nodule size is not significantly correlated with Bethesda cytopathology classification (50). Leong et al. argued that tumor size might be associated with the recurrence risk of high-cellularity subtype papillary thyroid carcinoma (51). Although Jasim et al. identified nodule location as an independent risk factor for predicting thyroid cancer, no studies have yet demonstrated a correlation between location and the benign or malignant nature of ITNs (52). Importantly, the proposed nomogram does not replace FNA, molecular testing, or clinical judgment. Instead, it provides an integrated risk assessment framework for patients who remain diagnostically challenging after FNA. For example, in patients with indeterminate cytology and equivocal molecular results, the combined model may provide additional imaging-derived evidence to support individualized decision-making. Conversely, patients with low predicted risk may be candidates for closer surveillance or repeat evaluation rather than immediate diagnostic thyroidectomy, depending on clinical context and patient preference.

This study has several limitations. First, being a retrospective study, it is subject to selection bias and data imbalance. Because surgical pathology was used as the reference standard, only surgically treated patients were included. This may have introduced selection bias and may partly explain the relatively high proportion of malignant nodules in the study cohorts. Future studies including patients managed with surveillance and long-term follow-up are needed to further evaluate the generalizability of the model. Second, due to the limited sample size for certain pathological types, the model currently cannot perform multi-class prediction of pathological outcomes. Larger sample sizes are needed in the future to build a multi-class model capable of classifying pathological grades. Third, elastography levels were assigned by a single experienced radiologist per examination without formal interobserver agreement calculation, which may introduce subjectivity. Although the grading followed a standardized scale, future multicenter studies should assess interobserver reliability for elastography. Besides, the manual ROI segmentation and complex radiomics pipeline used in this study may limit clinical feasibility. Nevertheless, the high reproducibility (ICC >0.8) and the potential for automated segmentation using deep learning suggest that the workflow can be streamlined. Future studies should focus on developing fully automated tools to reduce time and operator dependence.

## 5 Conclusions

In conclusion, our study demonstrates that features from two ultrasound modalities combined with genetic testing information can serve as biomarkers for constructing an online nomogram model to predict the malignancy of indeterminate thyroid nodules. This model helps reduce the need for repeat FNA or unnecessary thyroidectomy.

## Data Availability

The original contributions presented in the study are included in the article/**Supplementary Material**. Further inquiries can be directed to the corresponding authors.
