# Supplementary material for: An online nomogram based on bimodal ultrasound images for preoperative diagnosis of cytologically indeterminate thyroid nodules
Source: Front Endocrinol (Lausanne). 2026 Jun 29;17:1838372. doi: 10.3389/fendo.2026.1838372 (PMC13357161; doi:10.3389/fendo.2026.1838372)
Supplement: Supplementary file 1 [file DataSheet1.docx]

**Title:**

An Online Nomogram Based on Bimodal Ultrasound Images for Preoperative Diagnosis of Cytologically Indeterminate Thyroid Nodules

**Supplementary S1. Patient Inclusion and Exclusion Criteria**

All retrospectively collected thyroid nodule patients were enrolled according to the following criteria. The inclusion criteria for the study were: (1) patients with complete clinical data; (2) patients who underwent FNA examination with genetic testing; (3) nodules ultimately confirmed by pathological results from diagnostic or surgical resection; (4) ultrasound examination performed within two weeks prior to FNA. The exclusion criteria were: (1) history of other neoplastic diseases; (2) presence of thyroid inflammation; (3) history of thyroid microwave ablation or surgical treatment prior to ultrasound examination; (4) cytopathological Bethesda classification of I, II, or VI; (5) poor-quality ultrasound images.

For the prospective study, the inclusion criteria were as follows: (1) patients providing informed consent; (2) patients with suspicious thyroid nodules identified after ultrasound examination; (3) FNA performed after ultrasound examination with subsequent cytological diagnosis and genetic testing; (4) patients undergoing surgery within two weeks after ultrasound examination; (5) availability of clinical data. The exclusion criteria included: (1) history of other malignancies; (2) history of radioactive iodine ablation or thyroid surgery prior to ultrasound examination; (3) presence of thyroid inflammation; (4) cytopathological Bethesda classification of I, II, or VI.

**Supplementary S2. Ultrasound and FNA Examination Process**

B-mode and strain elastography ultrasound images were collected from two hospitals using ultrasound machines including Mindray Resona 7S, GE Volusion E10, HITACHI ALOKA, and SAMSUNG RS80A. All images were stored in the Picture Archiving and Communication System (PACS) workstation, and underwent preprocessing to minimize inter-device variability.

The representative equipment and settings were as follows: Samsung RS80A with an L3-12A linear-array transducer, depth 5.0 cm, MI 1.3, TIs 0.1, 2D gain 49, dynamic range 56, and frame average 4; GE Voluson E10 with a high-frequency linear-array transducer, small-parts/thyroid preset, depth 3.2 cm, MI 1.3, TIs/TIb 0.1; ALOKA ultrasound system with a 5415 probe, small-parts preset, depth 7.5 cm, gain 64, dynamic range 75, and SEUS frequency 21 Hz with a displayed quality indicator of 85%; and Mindray Resona 7S with an L14-5WU linear-array transducer, frequency 6.2–10.8 MHz, depth 3.0 cm, gain 63, frame rate 20 Hz, dynamic range 140, MI 1.1, TIS 0.0, and SEUS settings including Map E4, opacity 4, dynamic range 2, ESen 3, and SMode 1. Minor adjustments of gain, depth, and focus were permitted according to lesion depth and patient habitus.

The ultrasound examinations were performed by radiologists with over five years of experience. Patients were positioned supine with the neck fully exposed, and the thyroid gland was examined using a transducer perpendicular to the skin. Patients were instructed to hold their breath while the images were captured. Dynamic images of the lesion were retained, and strain elastography was performed on the maximum diameter cross-section of the nodule. The sampling box for elastography was as large as possible to fully include the lesion. After selecting the sampling box, the transducer was compressed (with slight pressure) and relaxed twice per second. Elastography levels were assigned using the 4-point scale developed by Asteria et al.: Level 1 indicated the nodule was mostly green with some red; Level 2 indicated light green and red within the nodule, with some blue around the periphery and center, predominantly green; Level 3 indicated mostly blue with some green and red within the nodule; and Level 4 indicated the nodule was entirely blue (1). Colors/ levels were considered stable if they remained consistent for 5-10 seconds over four repetitions.

For each thyroid nodule, a radiologist retrieved all available B-mode and strain elastography image series from the PACS. The following selection criteria were applied: (1) the image displaying the largest cross-sectional diameter of the nodule was prioritized; (2) from the same view, the single frame with optimal image quality (sharp nodule margins, absence of breathing or motion artifacts, and stable elastography color pattern for at least 5 seconds across four compression cycles) was selected; (3) if multiple nodules were present in one patient, images were selected separately for each nodule. All selected images underwent subsequent preprocessing and feature extraction. This selection process ensured that only high-quality, representative images were used for model development.

FNA was performed by physicians with more than 10 years of experience in ultrasound-guided interventions, using a 23-gauge fine needle under ultrasound guidance. The capillary technique was used to minimize blood contamination. A multi-spot puncture method was employed: three different sites within the nodule were punctured, and at each site, the needle was moved back and forth 10–20 times under capillary action without applying negative pressure. A portion of the aspirated tissue was placed on slides, immediately fixed with alcohol, and stained with hematoxylin and eosin. The remaining aspirated tissue was preserved in a cell preservation solution. DNA and RNA were extracted using the AllPrep DNA/RNA Micro Kit (Qiagen) and libraries were prepared for multiplex PCR target enrichment. Library sequencing was performed on the Novaseq 6000 (Illumina) using the 150 bp Pair-End sequencing mode. The analysis covered the target regions of the aforementioned genes, including those classified as tumor variants in the COSMIC database. Alignment and variant calling were performed using BWA (version 0.7.5a) and the Genome Analysis Toolkit (version 3.8). Variants were reported according to the Human Genome Variation Society (HGVS) nomenclature (http://www.hgvs.org/mutnomen/) and the human genome reference sequence GRCh37 (http://www.ncbi.nlm.nih.gov/refseq/rsg/). The coverage of the target regions was 100%. The kits, probes, and sequencing analysis were carried out by a laboratory (KingMed Diagnostics, Hefei, China), with specific procedures and result interpretations following the manufacturer's instructions. Negative and positive controls were included in each batch of tests. Commonly tested genes included BRAF, RAS, and TERT. Patients were classified into the positive group if one or more genes were found to be mutated.

**Supplementary S3. Image Feature Extraction Process**

**Step 1: Manual extraction of radiomic features.**

First, ROI 1 was manually delineated on the left side of the B-mode ultrasound (BMUS) image. For each nodule, after delineating ROI 1 on the BMUS image, we obtained ROI 2 on the SEUS image by reflecting the mask horizontally across the central dividing line. The extraction process was standardized according to the Image Biomarker Standardization Initiative (IBSI) (2). Radiomic features of the ROI were extracted using the Pyradiomics library in Python.

Features were extracted from both B-mode and strain elastography ultrasound modalities. For each modality, radiomics features were extracted using the Pyradiomics library with customized settings (detailed configuration provided in the supplementary settings file). The feature categories included shape2D, firstorder, gray-level co-occurrence matrix (GLCM), gray-level run length matrix (GLRLM), gray-level size zone matrix (GLSZM), gray-level dependence matrix (GLDM), and neighboring gray tone difference matrix (NGTDM). Both Original and Wavelet image types were used. Customized extraction parameters included normalization with a scale of 100, bin width of 5, B‑spline interpolation with resampled pixel spacing of [0.2, 0.2, 0], 2D force mode enabled, and a voxel array shift of 300.

The ROI delineation followed standardized criteria: (1) the ROI covered the entire nodule visible on the maximum diameter cross-section, including any internal cystic or necrotic areas (as these were not excluded due to indistinct boundaries); (2) the ROI boundary was traced along the visible interface between the nodule and the surrounding thyroid parenchyma; (3) for nodules with irregular margins, the delineation was based on the best estimate of the nodule border using overall shape and contrast difference; (4) all delineations were performed by a single radiologist (≥5 years of experience), and inter‑observer agreement was assessed using ICC > 0.8 on 50 randomly selected cases.

**Step 2: Extraction of deep features from both modalities.**

Based on ROI 1 and 2, grayscale and elastography images were cropped for transfer learning. The cropped square images encompassed the ROI, ensuring that both BMUS and SEUS images focused on the lesion area. DenseNet201 was used for training the transfer learning model. DenseNet201 has shown excellent performance in various image processing tasks, including image classification, object detection, and semantic segmentation (3, 4). A key feature of DenseNet201 is its densely connected design, where each layer directly receives feature maps from all preceding layers, significantly alleviating the vanishing gradient problem, promoting feature reuse, and reducing the number of parameters. DenseNet201 consists of 201 weighted layers, including convolutional and fully connected layers. Each convolutional block comprises multiple 3x3 convolutional layers followed by a 2x2 max-pooling layer, and this sequence is repeated to create a dense block. This design allows DenseNet201 to efficiently extract image features while maintaining compactness and high performance.

Given the limited dataset size, training a domain-specific classifier from scratch was challenging. To improve model performance and generalization ability, we utilized an ImageNet pre-trained model as the source model. ImageNet is a large-scale hierarchical image database that helps models learn valuable features and knowledge (ImageNet: A Large-Scale Image Database) (5). The weights of the pre-trained model were frozen, except for the final fully connected layer, which was replaced with a new layer initialized with random weights and subsequently trained to adapt to the specific classification task. The deep convolutional neural network (DCNN) was then trained using dual-modality images from the training set to predict the benign or malignant nature of indeterminate thyroid nodules. To extract deep features, all ultrasound images were fed into the hierarchical convolutional framework of the DCNN. Multiple feature maps were extracted and selected from the activation layer of the last convolutional layer, which has a dimension of 128. These feature maps were then quantified into statistical characteristics as deep features. We performed deep feature extraction on the cropped BMUS and SEUS images. The specific process is as follows:

First, training set images were preprocessed by resizing them to 224×224 and applying data augmentation techniques, including image scaling, translation, normalization, and contrast adjustment. To reduce overfitting and improve the performance of the convolutional layers, L2 regularization was applied to the final layer. The Adam optimizer was used with sparse categorical cross-entropy as the loss function for training the model. The learning rate was set to 0.0001, and the number of epochs was set to 100. If no improvement was observed after five epochs, the learning rate was reduced tenfold; if no improvement was observed after ten patience periods, training was stopped early. Finally, 128 deep features were extracted from each modality image. The deep feature extraction and transfer learning processes were implemented in Python (version 3.6.2) using TensorFlow (version 2.4.0) and Keras (version 2.4.3).

**Supplementary S4. Feature Selection and Scoring**

After feature extraction from all images, the radiomic and deep features from the same modality were fused. The intraclass correlation coefficient (ICC) was used to evaluate interobserver consistency, and radiomic features with ICC > 0.8 were retained. Z-score transformation was applied to standardize the feature data from both the training and test sets, adjusting the data to have a mean of 0 and a standard deviation of 1. This standardization helps reduce the impact of certain features with large raw values on the model and allows for comparison of different features on the same scale, avoiding variability in features that might arise from different protocols and operators. Statistical analysis was performed on the extracted feature data from the training set. Normality and homogeneity of variance tests were conducted first, and features with p > 0.05 were screened using the t-test to identify significant features. Features that did not pass the normality and homogeneity of variance tests were analyzed using the Mann-Whitney U test to identify significant features. The remaining features after t-test and U-test analyses were combined, and the Least Absolute Shrinkage and Selection Operator (LASSO) regression algorithm was used with fivefold cross-validation to select the optimal penalty coefficient lambda (λ). This process identified non-zero coefficient radiomic features in the training set. These non-zero coefficients were then weighted to derive the B-mode ultrasound score (BMUS score) and strain elastography ultrasound score (SEUS score) formulas. These formulas allow for the calculation of BMUS and SEUS scores for each patient, serving as predictors for indeterminate thyroid nodules (ITNs).

**Supplementary S5. BMUS and SEUS Feature Selection and Scoring Formulas**

A total of 464 radiomic features and 128 deep features were extracted from the ultrasound images of each modality. After random sampling and ICC analysis, features with ICC < 0.8 were removed, retaining 422 radiomic features for both ultrasound modalities. Next, the radiomic and deep features, totaling 550, were combined and standardized in the training set. The remaining features after t-test and U-test analyses were 421 for BMUS and 504 for SEUS. Finally, the LASSO regression algorithm was used to further refine the features and establish the scoring formulas. Fivefold cross-validation was employed to select the optimal penalty coefficient lambda (λ). As λ increased, the coefficients of each feature were gradually compressed to zero. In the end, 14 and 11 features were selected to construct the BMUS and SEUS scores.

The formulas for calculating the BMUS score and SEUS score are as follows:

BMUS score =

0.3626 + 0.4379 × original_firstorder_Skewness

+ 0.3519 ×original_shape2D_Elongation

-0.0523 × wavelet.LH_firstorder_10Percentile

- 0.1187 × wavelet.LH_firstorder_Skewness

- 0.0865 × wavelet.LH_glcm_MaximumProbability

+ 0.0711 × wavelet.LL_firstorder_Skewness

- 0.1224 × wavelet.LL_glcm_MaximumProbability

+ 0.2487 × Feature_15

- 0.1276 × Feature_40

+ 0.2679 × Feature_45

- 0.1583 × Feature_49

- 0.0439 × Feature_50

+ 0.0734 × Feature_100

- 0.0492 × Feature_126

SEUS score =

0.7065 + 0.6725 × original_firstorder_Skewness

- 0.8515 × original_gldm_LargeDependenceLowGrayLevelEmphasis

- 0.3312 × original_glszm_SmallAreaEmphasis

+ 0.6474 × original_shape2D_Elongation

- 0.9955 × wavelet.HL_ngtdm_Contrast

- 0.7708 × wavelet.LH_firstorder_Skewness

- 0.2582 × wavelet.LH_glrlm_LongRunLowGrayLevelEmphasis

- 0.5997 × wavelet.LH_glszm_GrayLevelNonUniformityNormalized

- 1.4520 × Feature_43

- 1.1689 × Feature_59

+ 1.6682 × Feature_103


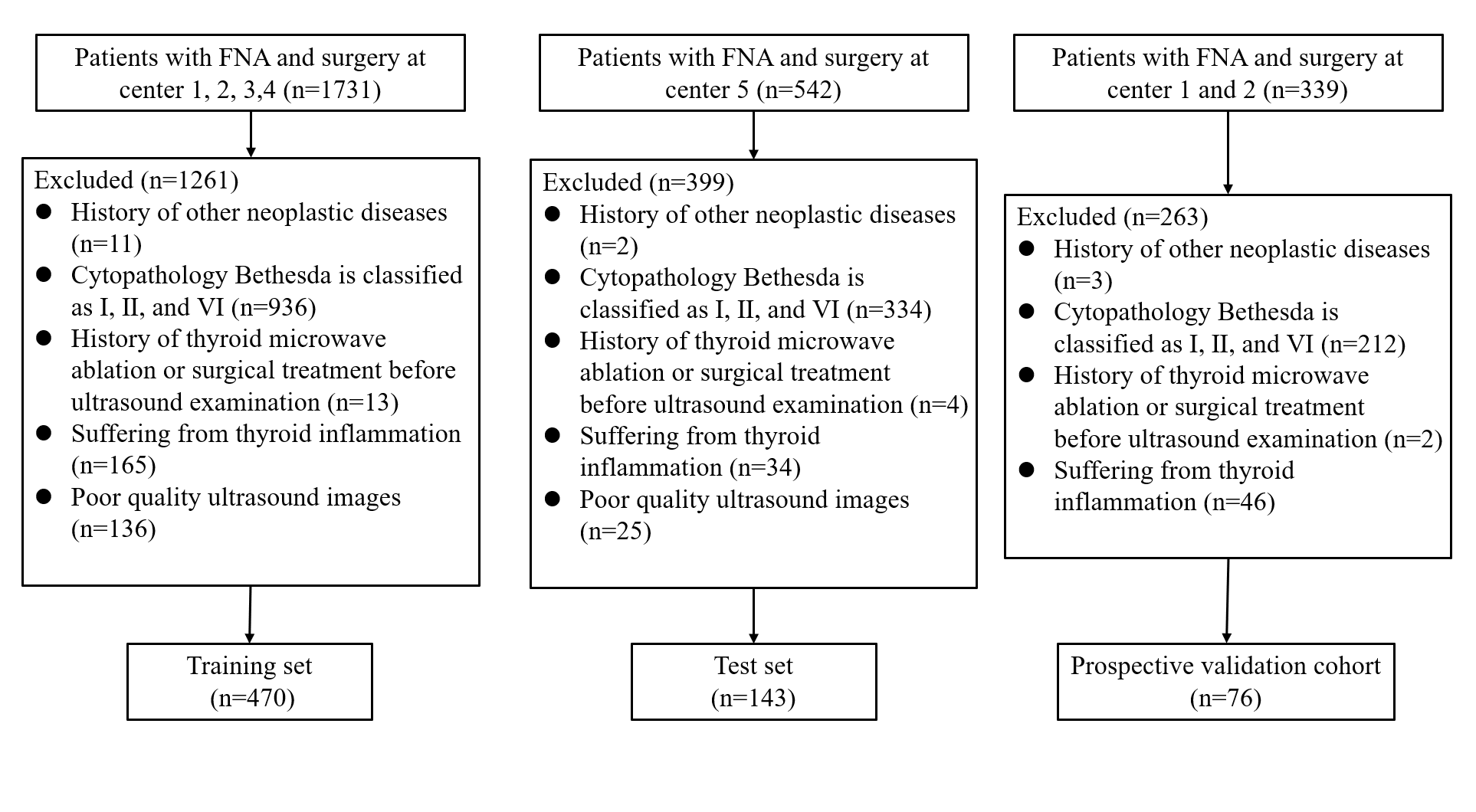


**Supplementary Figure S1.** Patient recruitment flowchart in this study.


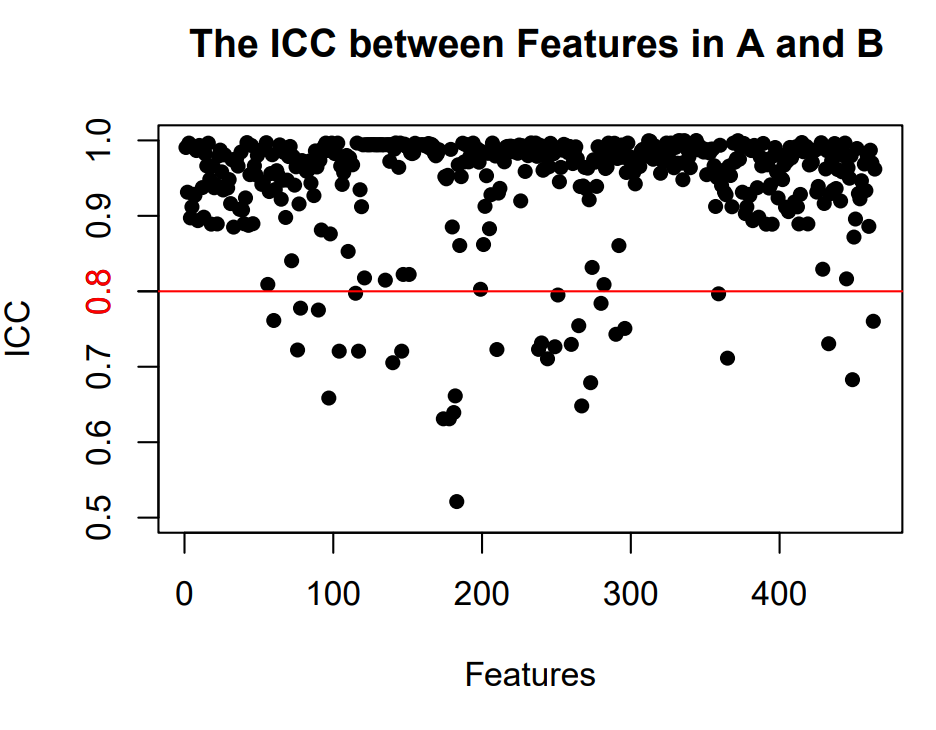


**Supplementary Figure S2.** Interclass Correlation Coefficient (ICC) between Doctors A and B. Features with an ICC value below the threshold of 0.8 were excluded.


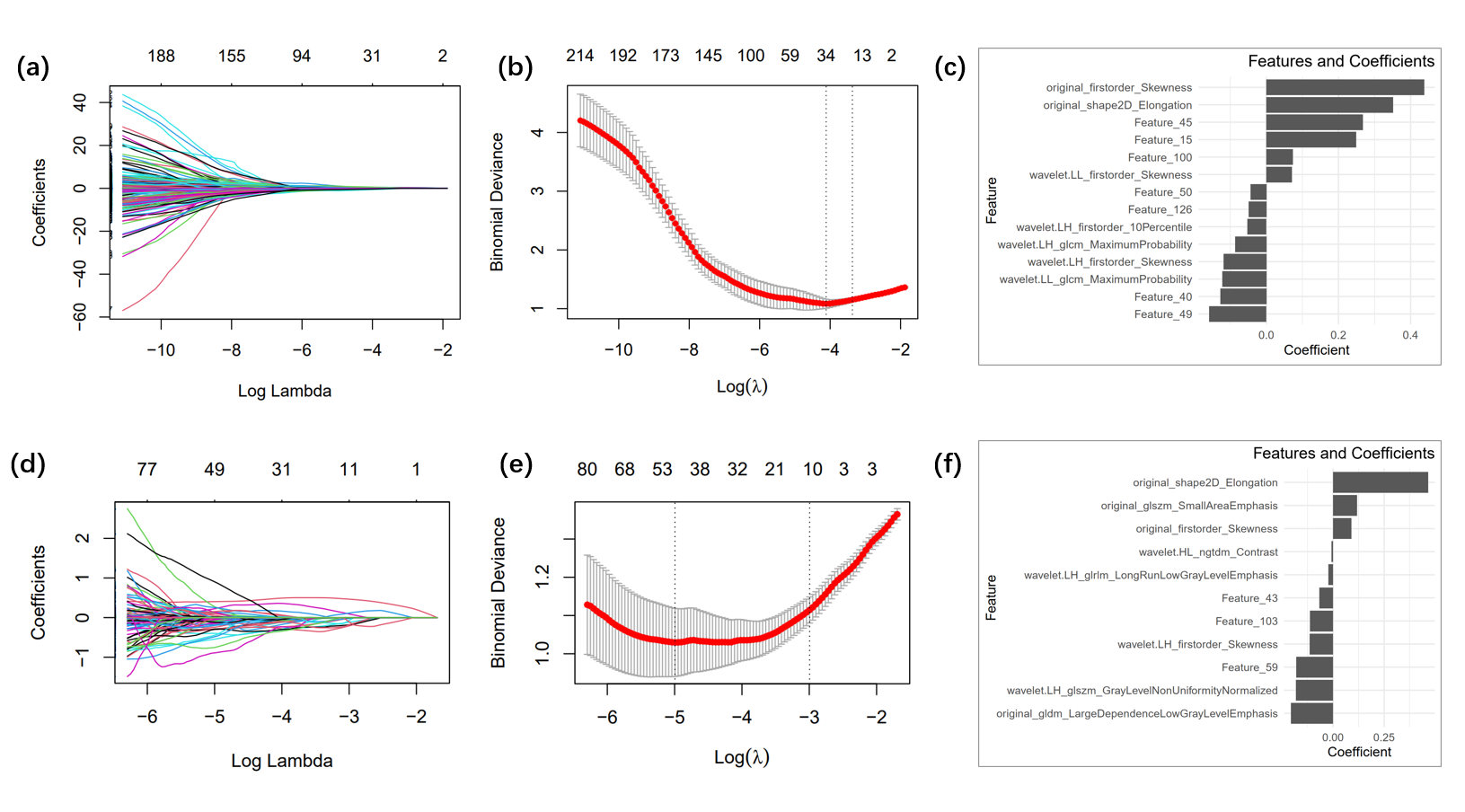


**Supplementary Figure S3.** Feature selection in the training set using the Least Absolute Shrinkage and Selection Operator (LASSO) algorithm. Panels **(a)** and **(b)** show the feature selection process for BMUS, while panels **(c)** and **(d)** show the process for SEUS. Panels **(e)** and **(f)** represent the feature composition of the BMUS score and SEUS score, respectively.

**References**

1. Asteria C, Giovanardi A, Pizzocaro A, Cozzaglio L, Morabito A, Somalvico F, et al. US-elastography in the differential diagnosis of benign and malignant thyroid nodules. Thyroid. 2008;18(5):523-31.

2. Zwanenburg A, Vallieres M, Abdalah MA, Aerts H, Andrearczyk V, Apte A, et al. The Image Biomarker Standardization Initiative: Standardized Quantitative Radiomics for High-Throughput Image-based Phenotyping. Radiology. 2020;295(2):328-38.

3. Meng M, Zhang M, Shen D, He G. Differentiation of breast lesions on dynamic contrast-enhanced magnetic resonance imaging (DCE-MRI) using deep transfer learning based on DenseNet201. Medicine (Baltimore). 2022;101(45):e31214.

4. Sanghvi HA, Patel RH, Agarwal A, Gupta S, Sawhney V, Pandya AS. A deep learning approach for classification of COVID and pneumonia using DenseNet-201. Int J Imaging Syst Technol. 2022.

5. Russakovsky O, Deng J, Su H, Krause J, Satheesh S, Ma S, et al. Imagenet large scale visual recognition challenge. International journal of computer vision. 2015;115:211-52.
